# miR-140-3p is a potential differential biomarker in benign phyllodes tumors and fibroadenoma of the breast

**DOI:** 10.1186/s12905-022-01613-4

**Published:** 2022-02-05

**Authors:** Qiang Li, Yao Liu, Jiajia Bai, Song Zhao, Yawen Wang, Arzoo Shabbir, Kai Zhang, Fan Jiang, Jiang Zhu, Rong Ma, Jianli Wang

**Affiliations:** 1 Department of Breast Surgery, General Surgery, Qilu Hospital, Cheeloo College of Medicine, Shandong University, Jinan, 250012 Shandong China; 2 Department of Pediatric Surgery, The Second Affiliated Hospital of Xi’an Jiaotong University, Xi’an, 710004 Shaanxi China; 3 Shandong Key Laboratory of Cardiovascular Proteomics, Qilu Hospital, Cheeloo College of Medicine, Shandong University, Jinan, 250012 Shandong China; 4 Department of Obstetrics and Gynecology, Qilu Hospital, Cheeloo College of Medicine, Shandong University, Jinan, 250012 Shandong China

**Keywords:** MicroRNA, Phyllodes tumor, Fibroadenoma, Biomarker, PCR

## Abstract

**Background:**

Benign phyllodes tumor (BPT) and fibroadenoma (FA) have some difficulties in differential diagnosis. BPT is often misdiagnosed as FA during the first operation and is not diagnosed until postoperative recurrence and reoperation. The intent of this research was to find and validate microRNAs (miRNAs) with significant differential expression between BPT and FA as novel potential differential biomarkers.

**Methods:**

Tissue specimens from three BPT patients and three FA patients were selected to detect the expression of miRNAs by miRNA-Seq technique. Primary cells were extracted and cultured from fresh BPT and FA tissues by tissue-block culture. The expression of differentially expressed miRNA (DEmiRNA) was further verified by quantitative real-time polymerase chain reaction (qRT-PCR) in twelve BPT and eleven FA patient specimens as well as primary cells. Data with a *P* value < 0.05 were considered statistically significant.

**Results:**

The miRNA-Seq results showed totally six DEmiRNA were identified, consisting of two downregulated genes and four upregulated genes in BPT. Further validation by qRT-PCR manifest that miR-140-3p was downregulated by approximately 70% in BPT.

**Conclusion:**

miR-140-3p could become potential differential biomarker for BPT and FA.

**Supplementary Information:**

The online version contains supplementary material available at 10.1186/s12905-022-01613-4.

## Introduction

Phyllodes Tumor (PT) accounts for about 0.3–1% in all primary breast tumors [1, 2]. Usually it occurs in females aged 40–50 years [3]. PT is classified as benign, borderline and malignant according to histological features, of which the most common is benign phyllodes tumor (BPT), accounting for approximately 60–75% of all phyllodes tumors [4]. Fibroadenoma (FA) of the breast is a heterogeneous group of bidirectionally-differentiated tumors with proliferation of both epithelial and stromal components in young women, which belongs to fibroepithelial tumors together with PT. It has been reported in the literature that there are similar gene mutations in PT and FA [5], so some scholars believe that FA can be converted into PT. However, FA does not actually contain real tumor cells as PT does; therefore, they are two diseases of different types.

Borderline PT and malignant PT are clearly distinguished from FA, while BPT and FA have similar clinical, imaging, and cytopathological findings [6]. Existing diagnostic methods such as ultrasound, molybdenum target, nuclear magnetic resonance imaging and needle biopsy are difficult to provide accurate diagnostic results [7]. Complete resection of the tumor for pathological examination is the gold standard for differentiating BPT from FA. Clinically, FA may achieve local control with simple mass resection, while PT requires local extensive resection [8]. Most studies suggest that benign PTs require at least a tumor-free margin of more than 1 cm to prevent recurrence [9, 10]. Because the vast majority of benign PTs (87%) have areas similar to FA on pathological sections [11], BPT is easily misdiagnosed as FA and performed with lumpectomy, and the correct diagnosis is not made until the pathological section is reviewed for recurrence after surgery. Moreover, BPT may transform into borderline and even malignant phyllodes tumors after recurrence, and distant metastasis leads to patient death [12], so there is a need to find new and reliable tumor biomarkers for differentiating BPT from FA.

MicroRNAs (miRNAs) are an evolutionarily conserved class of endogenous single-stranded non-coding small RNAs of approximately 22 nucleotides in length that account for approximately 3% of the human whole genome [13]. miRNAs play an important part in tumor development mainly by degrading target mRNAs or inhibiting the translation of target proteins [14, 15]. Detection of single miRNA or miRNA expression profiles in serum or biopsy tissue can be available for the differential diagnosis of diseases. A few studies have reported that miRNAs can act as differential biomarker of benign and malignant PT [16, 17], but there is no relevant report on miRNA expression in BPT and FA.

Several published articles have reported that miR-140-3p plays an important part in a variety of diseases. miR-140-3p can act as potential biomarker for osteoarthritis, autism spectrum disorder, osteoporosis and osteoporotic fractures [18–20]. In cancer, miR-140-3p usually acts as cancer suppressor, such as suppressing cell growth in colorectal cancer by PD-L1, suppressing growth and invasion of cell lung cancer by down-regulating ATP8A1, and inhibiting progression of cutaneous melanoma by targeting ABHD2 [21–23]. The intent of this study was to find molecular markers that can be used for differential diagnosis by screening and validating DEmiRNAs in FA and BPT.

## Methods

### Ethics approval and consent to participate

The written informed consent of each patient participating in the study was obtained. The study protocol and informed consent were approved by the Ethical Committee of Qilu Hospital of Shandong University. All of the methods were carried out in accordance with the Declaration of Helsinki.

### Sample collection and preparation

All 25 specimens required for this study were obtained from patients admitted to the Department of Breast Surgery, Qilu Hospital of Shandong University for surgical treatment from January 2017 to January 2021. After surgery, all of the tumors were subjected to a histopathologic diagnosis by two pathologists. Due to the small sample size, we only grouped the samples by pathology. All tissues used in the experiments were stored in liquid nitrogen after surgical resection.

### Extraction of RNA

Briefly, an appropriate amount of tissue was cut from the specimen and 1 ml of Trizol (Ambion, USA) was added and ground in a grinder until flocculent. The ground tissue was subjected to subsequent procedures according to the trizol method. The concentration of RNA was measured and adjusted to 200–500 ng/μL for subsequent manipulation.

### Human miRNA-seq and date analysis

The Human miRNA-seq was performed by KangChen Biosciences (China). Detailed steps are provided in Additional file 1.

### Quantitative real-time polymerase chain reaction (qRT-PCR)

One thousand ng of total RNA from each sample were reverse transcribed by PrimeScriptTMRT regent Kit with gDNA eraser (TaKaRa, USA) with a final reverse transcription primer concentration of 0.25 μM. First, reagents 1 and 2 were added to each reaction system according to the instructions, and the total amount of liquid was supplemented to 10 μL by dd H_2_O and heated at 42 degrees for 2 min to remove gDNA. Then, reagents 3 and 4 and reverse transcription primers were added, and dd H2O was added to make the total liquid volume of each reaction system reach 20 μL. Set the heating conditions to 42 degrees for 15 min and 85 degrees for 5 s. The resulting cDNA was subjected to PCR in a Step One real-time PCR machine (ABI, USA) following the instructions of the FastStart Essential DNA Green Master (Roche, USA), and a melting curve was drawn. The final concentration of PCR primers was 0.3 μM. miR-10b-5p primers (Biosune Biotechnology, China) for PCR were: 5′‐GGCGAAGAGTACCCTGTAGAACC‐3′ (sense) and 5′‐TATGGTTGTTCACGACTCCTTCAC‐3′ (antisense). miR-126-5p primers (Biosune Biotechnology, China) were 5′‐TGGTGGAGGCATTATTACTTTTGG‐3′ (sense) and 5′‐GTGCAGGGTCCGAGGT‐3′ (antisense). miR-140-3p primers (Biosune Biotechnology, China) for PCR were: 5′‐TCACTGCTCTGTACCACAGGGTAG‐3′ (sense) and 5′‐ TATGGTTTTGACGACTGTGTGAT‐3′ (antisense). U6 primers (Biosune Biotechnology, China) were 5′‐CAGCACATATACTAAAATTGGAACG‐3′ (sense) and 5′‐ACGAATTTGCGTGTCATCC‐3′ (antisense). Relative expression level was computed using 2^−ΔΔCt^ method.

### Primary cell culture

Primary cells were extracted by tissue-block culture method: small pieces of fresh tissue were cut and washed with sterile PBS for 3 times; surface fat and fascia were removed, cut to small pieces with a diameter of about 1 mm, and spread flat to the bottom of culture flask, with the distance between small pieces of tissue being about 0.3 cm; the flask was turned upwards, an appropriate amount of culture medium was added, and the culture flask was placed in a constant temperature incubator (37 °C, 5% CO2). After cultured for 2–4 h, the flask was slowly turned over after the small pieces dried up. Cell sprouting can be observed after 24 h.

### Statistical analysis

All data from this experiment were analyzed by GraphPad Prism 7 (GraphPad Software, USA). For each sample, RNU6 was used as an internal reference to calculate the Δct value of each miRNA separately. The Δct values of each miRNA in the FA group were averaged and used as the control group to calculate the ΔΔct values of each sample, respectively. Finally, the relative expression level of each miRNA for each sample was obtained by calculation (value = 2^ (−ΔΔct)) and analyzed with this data. Expression levels of each sample from different groups were statistically analyzed by unpaired t-test, and the data with *P* value < 0.05 in the statistics were considered statistically significant.

## Results

**Fig. 1:**
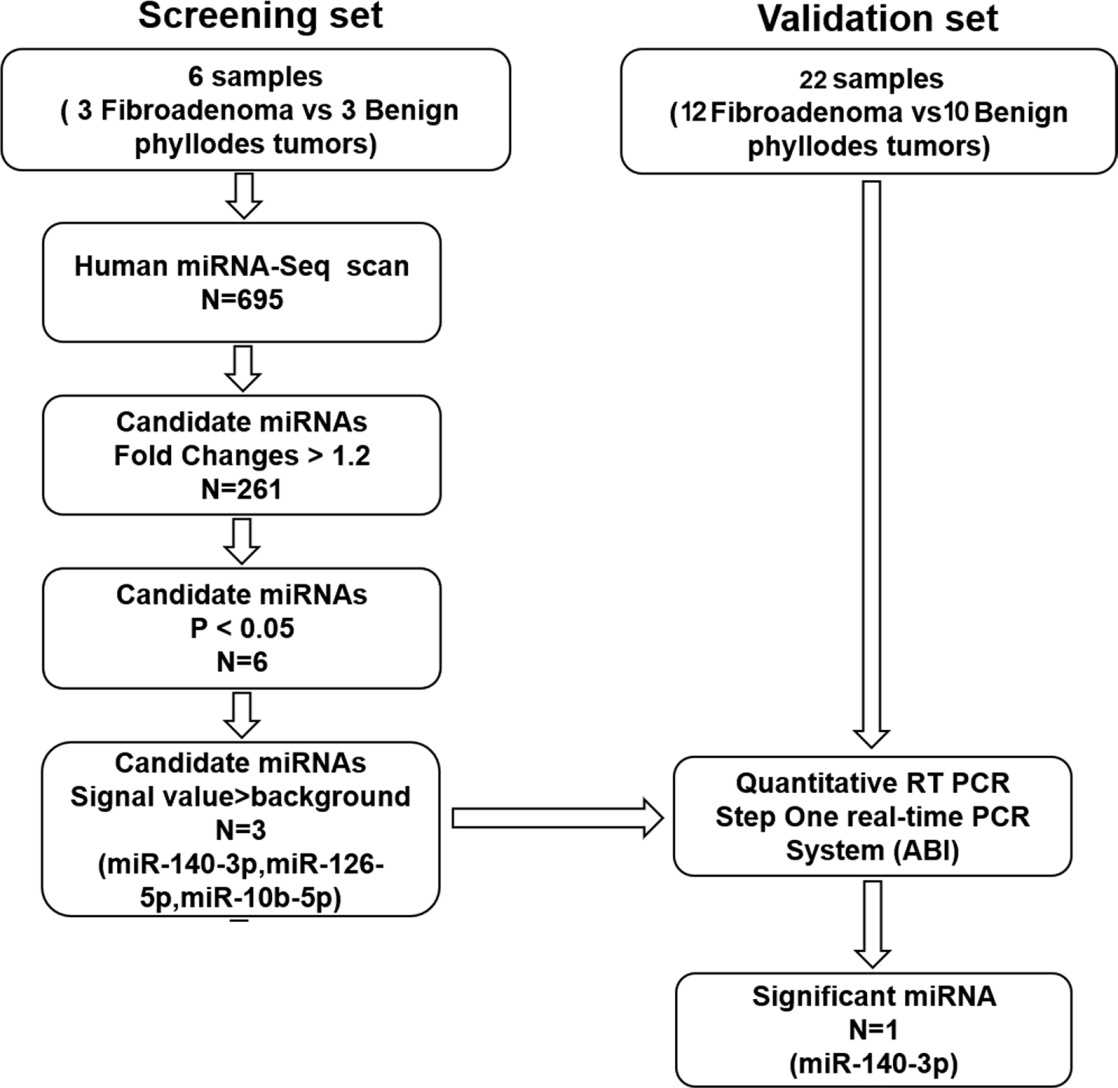
Study design flowchart. Human miRNA-Seq was used for screening candidate miRNAs, and qRT-PCR was used for validation

**Fig. 2:**
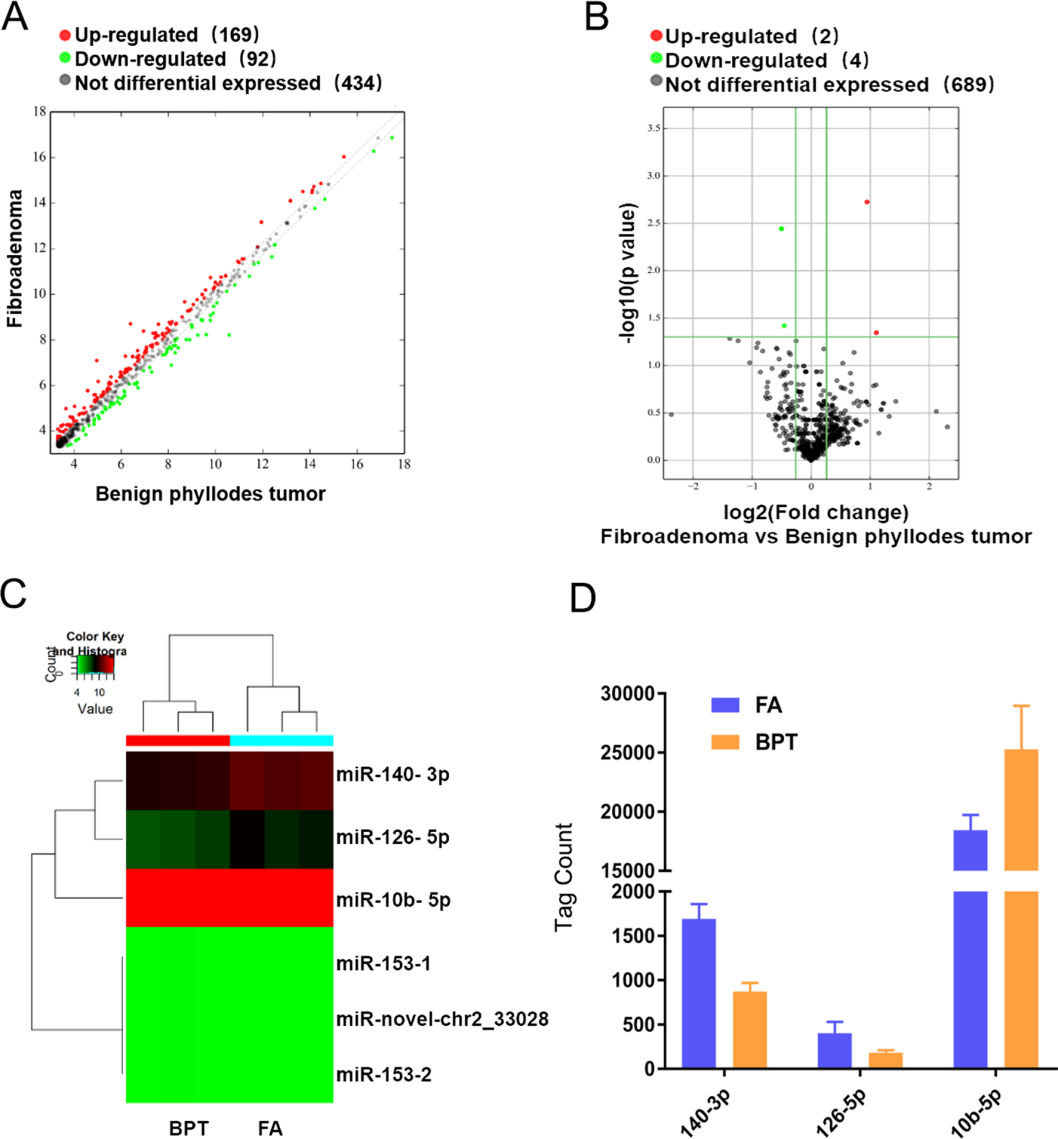
Expression of differentially expressed genes (DEmiRNA) between fibroadenoma (FA) and benign phyllodes tumor (BPT). **A** Scatter plot show DEmiRNA between two groups. Red dots represent up-regulated miRNAs, green dots represent down-regulated miRNAs and gray dots represent miRNAs without differential expression (fold change > 1.2). **B** Volcano plot shows DEmiRNA with p-valued between two groups (*P* < 0.05 was considered significant). **C** Heat map diagram showing hierarchical clustering of differentially expressed miRNAs between two groups. The color scale shown at the top illustrates the relative expression of miRNAs, with red for relatively high and green for relatively low expression. d**.** Tag count of three DEmiRNA in two groups

**Fig. 3:**
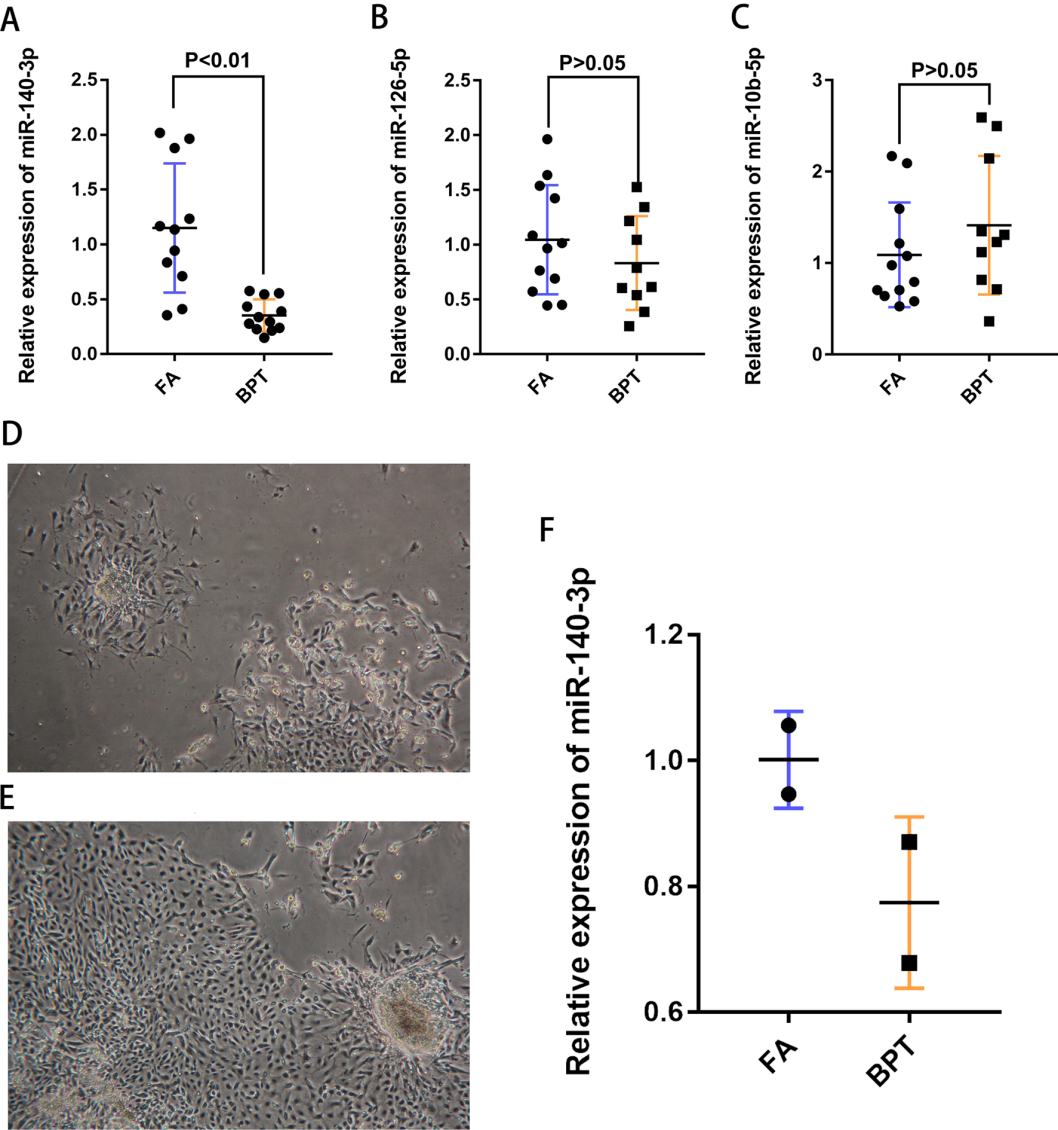
Validation of selected miRNAs in samples of FA and BPT. **A**–**C** The level of selected miRNAs in each sample was normalized to the internal control, RNU6, and shown as the relative expression. **A** miR-140-3p, **B** miR-126-5p, **C** miR-10b-5p (*P* < 0.05 was considered significant). **D** Primary cell of FA (40 × magnification). **E** Primary cell of BPT (100 × magnification). **F** Relative expression level of miR-140-3p of primary cell from two groups

The experimental design of this study is shown in Fig. 1. First, tumor tissues from three FA and three BPT patients were selected to detect the expression levels of miRNAs by miRNA-seq, and the differential expression of miRNAs were analyzed. Among the 695 miRNAs, 261 differentially expressed genes (DEmiRNA) with fold changes greater than 1.2-fold were selected, of which 169 miRNAs were up-regulated and 92 miRNAs were down-regulated, and the scatter plot showed the miRNA expression between the two groups (Fig. 2A). Six of these miRNAs had significant differences in expression (Fig. 2B). Compared with FA, miR-140-3p and miR-126-5p in BPT showed down-regulation, and miR-10b-5p, miR-153-3p (with two pre-miRNAs, mir-153-1 and mir-153-2) and miR-novel-chr23028 showed up-regulation (Fig. 2C). Among them, the tag counts of these two miRNAs, miR-153-3p and miR-novel-chr233028, in the miRNA-seq were close to the background, so only the three miRNAs, miR-140-3p, miR-126-5p and miR-10b-5p, were studied, and their tag counts are shown in the figure (Fig. 2D). Meanwhile, ten BPT and twelve FA tissues were selected for RNA extraction and qRT-PCR was performed to validate the screening results. Through validation, it was found that although the differential trends of miR-140-3p, miR-126-5p and miR-10b-5p were consistent with the gene chip results, only the difference in the expression of miR-140-3p was statistically significant between the two groups. The miR-140-3p expression level was down-regulated by 70% in the PT group compared with the FA group (Fig. 3A–C).

Primary cells were extracted by tissue-block culture from two FA and two BPT tissues (Fig. 3D, E), and the expression levels of miR-140-3p of both cells were detected by qRT-PCR. The results showed that the expression level of miR-140-3p in FA cells was also higher than that in BPT cells (Fig. 3F), which was the same as the differential expression between tissues.

## Discussion

miRNAs play an important role in tumor development by regulating the expression and activity of downstream target genes or other signaling molecules. Upregulated or down-regulated miRNAs may result in the suppression of the expression of oncogenes or tumor suppressor genes, suggesting that miRNAs may serve as both tumor suppressor genes and oncogenes [24]. In recent years, many teams have carried out related research on miRNAs in disease diagnosis and treatment. A variety of malignant epithelial tumors including colon, breast, prostate, and pancreatic cancers can be diagnosed early by detecting changes in the expression of a single miRNA or miRNA profile in serum. In terms of differential diagnosis, miR-K12-5-5p, miR-4732-5p, miR-4484, and miR-3646 were differentially expressed in nipple discharge between control and breast cancer [25]. MiR-221-5p and miR-708-3p are down-regulated in prostate cancer compared with benign prostatic hyperplasia and can be used for the diagnosis of prostate cancer with normal PSA [26]. The ratios of miR-15b-5p, miR-20a-5p, miR-19a-3p, miR-92a-3p and miR-16-5p to internal reference miR-146b-3p in serum are differentially expressed in patients who suffer from benign or malignant lung nodules and can be used to differentiate squamous cell lung cancer and benign lung diseases [27]. There are few studies on miRNAs associated with PT, and it has been reported that miR-21, miR-155 and miR-335 are over-expressed in malignant PT, which promotes the development of malignant PT [16, 17]. Therefore, we suggest that miRNAs also have a role in differentiating FA from BPT.

It is important to find miRNAs as potential differential diagnostic biomarkers for BPT and FA. In this study, we screened six miRNAs with fold changes greater than 1.2 with statistically significant among 695 miRNAs by miRNA-Seq technology, of which two miRNAs were down regulated (miR-140-3p and miR-126-5p) and four miRNAs were up regulated (miR-10b-5p, miR-153-3p (mir-153-1 and mir-153-2) and miR-novel-chr233028) in BPT. By observing the tag count, three miRNAs with higher expression content were selected for validation. Compared with FA patients, the expression level of miR-140-3p in tissues from BPT patients was significant decrease. It can be found by scatter plot that the expression within the BPT group was stable at a lower level, while the miR-140-3p expression level within the FA group fluctuated greatly, but almost all were higher than those in the BPT group were. We believe that due to some similarity between FA and BPT in organization, the miR-140-3p expression level in some FA is close to BPT, but the level of miR-140-3p in most FA is still higher than BPT. In validation set, the difference trend of miR-126-5p and 10b-5p between BPT and FA groups was the same as that of the screening group, but the expression levels of them in tissues were unstable, and it was difficult to determine a boundary to distinguish the two diseases. Due to the lack of a sufficient number of BPT specimens, intragroup differences are reduced by expanding the sample size or setting grouping conditions. Meanwhile, primary cells were extracted from 2 FA and 2 BPT tissues, respectively, and the expression levels of miR-140-3p were detected, and it was found that cells also showed the same difference situation as tissues. Both screening and validation results showed that the expression level of miR-140-3p was significantly different in FA and BPT, while the differential expression was the same between cells and tissues, and the expression level of miR-140-3p was stable in BPT, so we concluded that miR-140-3p could serve as a potential biomarker to differentiate FA from BPT.

In order to establish a miRNA as a biomarker for differentiating FA from BPT in clinic, the following issues need to be solved. First, to find a miRNA with significant expression differences between FA and BPT, this study demonstrated that the expression of miR-140-3p is significantly different between FA and BPT through screening and validation, and the potential mechanism of the effect of this miRNA on the development of BPT still needs further study. Second, from the validation results, it can be seen that the expression of miR-140-3p is more stable in tumor tissues of BPT patients, but the expression fluctuates greatly in FA patients, and it is still necessary to expand the sample size to further verify the feasibility of miR-140-3p as a molecular biomarker. Finally, if miR-140-3p serve as a clinical differential index, a boundary or interval needs to be determined to distinguish the two diseases, FA and BPT, which has higher requirements for specimen volume as well as detection methods. When performing preoperative needle biopsy and intraoperative rapid frozen pathology, a marker with differential significance helps physicians make a correct diagnosis and differential diagnosis of the patient's condition, resulting in better treatment.

In summary, this study found and validated significant differences in the expression levels of miR-140-3p between FA and BPT patient tissues by screening. Compared with FA, miR-140-3p expression levels were down-regulated by about 70% in BPT patient tissues, and miR-140-3p expression levels were also decreased in BPT primary cells, and we concluded that miR-140-3p can be used as a potential molecular biomarker to differentiate FA from BPT, while miR-140-3p may regulate the development of BPT by targeting one or more target genes. The experimental results of this study have some guiding significance for studying the occurrence and development of FA and BPT.

## Supplementary Information

**Additional file 1.** Methods of Human miRNA-seq and Date analysis.

## Data Availability

The data that support the findings of this study are available from the corresponding author upon reasonable request.

## Abbreviations

PT: Phyllodes tumor
BPT: Benign phyllodes tumor
FA: Fibroadenoma
miRNAs: MicroRNAs
DEmiRNA: Differentially expressed genes
