# Supplementary material for: miR-140-3p is a potential differential biomarker in benign phyllodes tumors and fibroadenoma of the breast
Source: BMC Womens Health. 2022 Feb 5;22:31. doi: 10.1186/s12905-022-01613-4 (PMC8817532; doi:10.1186/s12905-022-01613-4)
Supplement: Supplementary file 1 — Additional file 1. Methods of Human miRNA-seq and Date analysis. [file 12905_2022_1613_MOESM1_ESM.docx]

**Supplementary Material1: Methods of Human miRNA-seq and Date analysis**

Total RNA of each sample was used to prepare the miRNA sequencing library, which included the following steps:

1) 3'-adaptor ligation;

2) 5'-adaptor ligation;

3) cDNA synthesis;

4) PCR amplification;

5) Size selection of ~135-155 bp PCR amplified fragments (corresponding to ~15-35nt small RNAs).

The libraries were denatured as single-stranded DNA molecules, captured on Illumina flow cells, amplified in situ as clusters and finally sequenced for 51 cycles on Illumina NextSeq per the manufacturer's instructions.

After sequencing, the Solexa CHASTITY quality filtered reads were harvested as Clean Reads. The adaptor sequences were trimmed and the adaptor-trimmed-reads (>= 15nt) were left. miRDeep2 software was used to predict the novel miRNAs with these trimmed reads. Then, the trimmed reads were aligned to merged pre-miRNA databases (known pre-miRNA from miRBase v21 plus the newly predicted pre-miRNAs) using Novoalign software (v2.07.11) with at most one mismatch. Reads (counts < 2) were discarded when calculating the miRNA expression. In order to characterize the isomiR variability, sequences that matched the miRNA precursors in the mature miRNAs region ±4 nt (no more than 1 mismatch) were accepted as mature miRNA isomiRs, which were grouped according to the 5-prime (5p) or 3-prime (3p) arm of the precursor hairpin. The numbers of mapped tags that were defined as the raw expression levels of that miRNA. To correct for the difference in tag counts between samples, the tag counts were scaled to TPM (the copy number of transcripts per million) based on the total number of tag aligned. Choosing a different isomiR sequence for measuring miRNA expression can affect the ability to detect differential miRNA expression. We use the most abundant isomiR, the mature miRNA annotated in miRBase and all isoforms of miRNA (5p or 3p) to calculate the miRNAs expression. When comparing the differentially expressed miRNA profiles between two groups, fold change and p-value were calculated and used to identify significant differentially expressed miRNAs (Based on ALL_Isoform value). Differentially expressed miRNAs between two samples were filtered through Fold change (Based on ALL_Isoform value). Hierarchical clustering was performed.
